# Marketing approaches for OTC analgesics in Bulgaria

**DOI:** 10.1080/13102818.2014.911477

**Published:** 2014-07-15

**Authors:** Valentina Petkova, Velislava Valchanova, Adel Ibrahim, Irina Nikolova, Niko Benbasat, Milen Dimitrov

**Affiliations:** ^a^ Faculty of Pharmacy, Medical University-Sofia, Sofia, Bulgaria

**Keywords:** OTC analgesics, market analysis, marketing strategies, SWOT analysis

## Abstract

The marketing management includes analysis of market opportunities, selection of target markets, planning, developing and implementing of marketing strategies, monitoring and result control. The object of the present study was to analyse the marketing approaches applied for non-steroidal anti-inflammatory drugs (NSAIDs) in Bulgaria. The performed SWOT(planning method used to evaluate the strengths, weaknesses, opportunities, and threats) analysis for one of the leading Bulgarian manufacturers marked the complex corporative strategy for stimulating the sales of NSAIDs. The study results show that the legislation frame in the country gives an opportunity for regulation of the NSAID market in order that incorrect marketing approaches such as disloyal competition are avoided.

## Introduction

Analgesics and non-steroidal anti-inflammatory drugs (NSAIDs) are the most commonly prescribed pharmaceuticals for alleviating the pain. The pain management industry was estimated to exceed $26 billion (bn) in total spend in 2005 with certain sub-verticals growing at greater than 20%. More significantly, prescription and non-prescription NSAID sales are estimated at $18 bn annually worldwide. The four major uses for over-the-counter (OTC) pain relievers are headache, sore muscles, arthritis and heart-attack prevention, and their use is increasing worldwide (3). The OTC NSAID US market is a mature segment exceeding $2 bn in annual sales. *In 2010 the OTC market in the Central European Region (CER) worth some €10.7 bn, which represented an increase of approximately 13% year-over-year in euro terms. There is a forecast that between 2011 and 2013 the market will expand by around 9% on average per annum. Bulgaria and Ukraine will be the fastest developing markets in the region in the analysed period.* Following the rapid expansion in Bulgaria's OTC medicine market between 2004 and 2008, where a compound annual growth rate (CAGR) of 16.8% was recorded in local currency terms, growth rates in 2009 dropped to just 3.2%. The reduction has a strong correlation with Bulgaria's economic downturn and associated reductions in consumer spending. As in many other European states, the economic downturn has affected OTC sales more than prescription medicines, owing to the less essential nature and lack of government reimbursement of OTC drugs. Bulgaria's OTC medicine market picked up in 2010, following a major deceleration in 2009.[1] 

In the whole world, as well as in Bulgaria, there is a trend towards self-medication. This is attributed to the clumsy health care systems. As a result, many patients initially try self-medication with OTC products from the pharmacy before consulting a physician. Thus, the responsibility for the health of the patient shifts from the physician to the patient himself. Analgesics and NSAIDs continue to be one of the most widely prescribed and used classes of drugs worldwide, with both prescription and OTC formulations available in Bulgaria.

According to Kotler,[2] marketing is a social process by which individuals and groups obtain what they need and want through creating and exchanging products and value with others. *The American marketing association defines it as* the activity, set of institutions, and processes for creating, communicating, delivering, and exchanging offerings that have value for customers, clients, partners, and society at large. Adcock [3] shortly describes it as sense *of* the right product, in the right place, at the right time and at the right price.

The term ‘marketing mix’ was coined in 1953 by Neil Borden in his American Marketing Association presidential address. The marketing mix (price, product, distribution and promotion) forms the entire promotional campaign.[4] McCarthy [5] proposed a ‘four P’ (price, product, place and promotion) classification in 1960 that since then has been widely used. The term describes all the actions which have to be taken by marketing experts for attraction and keeping customers. The rivalry in the business is based on the best created four Ps’ combination.

The proper marketing management consists of analysis of market opportunities, selection of target markets, planning, developing and implementing marketing strategies, monitoring and controlling of results. The main aspects of the pharmaceutical marketing are as follows:

1. Right product: it has to treat and improve disease/s. The pharmaceutical manufacturer must be sure that the drug that will be developed can be beneficial from one point of view, and from the other that there is a marketing place for it. Otherwise, no matter how effective and of high quality the drug will be, there is a risk not to reach the pharmacy and the patient;
2. Right quantity: the quantity characteristics of the drug are strongly related to the packaging of the drug, and from there to its accessibility and advertisement. This perception is value for the OTC drugs. As the prescription-only-medicine (POM) is concerned, the right quantity depends on the decision of the doctor and it is reflected in the prescription and in the prescribed dosage;
3. Right price: this marketing aspect is very important regardless the fact that the drugs are needed and that they will be purchased in order to treat a disease. In some cases when there is no acute disease, or most of the time the chronic disease is symptomless (like stage I of hypertension) the price and solvency of the patient determine the treatment;
4. Right time: this is a largely underestimated factor. If the advertisement of a new drug is not in conformity with the seasoning, then the marketing activity will fail to increase the purchasing rate.[6]

Analgesics are the most often dispensed drugs. In Bulgaria there are over 180 trade names of NSAIDs (as mono products or in various combinations, dosage and pharmaceutical forms) registered as OTC.[7] The aim of this study is to analyse the marketing approaches applied in Bulgaria by some of the NSAID manufacturers on the Bulgarian market in order to assess their marketing strategy.

## Materials and methods

The analysis of these issues is based on literature studies and the observation of practice on the Bulgarian pharmaceutical market. In the present paper the following groups of methods were applied:

1. Methods for collecting of primary information: market test, target group;
2. Comparative analysis of dynamics of sells (method of comparison);
3. Methods of collecting of secondary information: expertise and content analysis;
4. SWOT analysis.

The following groups of materials were analysed:

1. Regulatory status of NSAIDs in Bulgaria;
2. Business Monitor International reports;
3. Market of NSAIDs in volume and in value.

## Results and discussion

### Legislation review

The main pharmaceutical law regulating the drugs in Bulgaria is the Bulgarian Drug Law in forces since 2007. It classifies the drugs into two main groups – Rx and OTC drugs. The regimen of the drugs is determined by the Bulgarian Drug Agency (BDA) with the marketing authorization for use. The requirements for advertisement are regulated in Chapter 11 of the Law that is harmonized with Directive 2001/83/EU and Regulation No13. Advertising of medicinal products shall be any form of information, provision, promotion or offers aimed at stimulating the prescribing, sale or use of a medicinal product and shall include the following:

1. Advertising intended for the population;
2. Advertising intended for medical specialists;
3. Visits of medical representatives to health professionals;
4. Distribution of samples of medicinal products;
5. Sponsorship of promotional meetings and scientific congresses visited by health professionals.[8]

Expert Council of advertising draws expertise on advertisement project, it prepares an opinion to the executive director of BDA who authorizes or rejects the project. BDA monitors pharmaceutical promotion, TV and radio advertising (only for OTC drugs). Samples of medicinal products can be provided to physicians to get acquired with it and only to the extent that the concerned persons are qualified to prescribe the medicinal product, and are permitted to use the product as part of their activities. Their annual number is limited and every sample has to represent the smallest package in the market. The label ‘free sample’ is compulsory to be on the package.

A great part of the studied group of drugs is OTC and can be advertised directly to the population. For advertising marketing authorization holder (MAH) shall submit to the BDA a formal application as approved by the executive director of the agency accompanied by (1) design of the advertising; (2) notarized power of attorney issued by the MAH where the application shall be submitted by another person; (3) bibliographical references of the used quotations, tables, or other materials, if any; and (4) document for paid fee to the amount as paid down in the tariff.[8] It can be concluded that the promotion of drugs and especially of OTC is well regulated in Bulgaria. OTC status gives a possibility for direct to consumer marketing, which leads to sales and profit increase.[9] Most of the marketing campaigns for the NSAIDs in Bulgaria include product branding, concise information on the product throughout the mass media (TV, radio and print press advertising) as well as providing web branding through e-advertisement, a new way of promotion that is not included in the legislation frame so far. In Bulgaria there are active legislative measures that help for the loyal competition in this segment of the Bulgarian drug market.

### Characteristic of Bulgarian pharmaceutical market of NSAID

The deep recession, cut of reimbursement resources, market restrictions due to the population size as well as limited financial potential make the market situation really difficult. However, there is a tendency of production modernization and foreign investment in the industry resulting from European Union (EU) membership. Patented drugs market presently accounts about 52% of the total by value but generic drugs will continue to decrease its market share, stimulated by the lower costs of the latter. It is expected that the government will aim to shift more expenditure onto the patient resulting in the development of a stronger OTC segment, as consumer incomes and health awareness levels increase. With EU membership Bulgaria has been forced to become stricter in enforcing adherence to prescription rules and with it the OTC sector is likely to experience a significant boost in the future. Despite of being relatively underdeveloped in European terms, Bulgaria's OTC market has expanded rapidly over the past few years. Business Monitor International (BMI) forecasts that the OTC market will reach a value of BGN 574 mn (USD 334 mn) by 2014, increasing to BGN 629 mn (USD 365 mn) by 2019 (Table 1).[10,1]

**Table 1.** Marketing development of OTC drugs.[10,1]

	2005	2006	2007	2008	2009	2010	2011	2012
OTC market BGN bn	0.223	0.253	0.308	0.375	0.388	0.422	0.471	0.515
OTC share % from the whole market	18.72	19.70	20.90	22.01	21.00	21.80	22.70	23.20
Analgesics drug sales	35.0	40.1	46.2	58.0	62.8	61.0	60.6	63.40
	2013	2014	2015	2016	2017	2018	2019	
OTC market BGN bn	0.546	0.574	0.592	0.605	0.619	0.629	0.629	
OTC share % from the whole market	23.15	23.10	22.80	22.5	22.40	22.35	22.22	
Analgesics drug sales	65.3	65.2	65.1	64.5	63.9	62.7	60.4	

According to the pharmaceutical market research, the analgesics category was valued at BGN 88.8 mn ($66.4 mn) in 2009, representing a *CAGR* of 11.6% since 2004. By the end of 2014, the analgesics category will be worth BGN 125.3 mn ($93.7 mn), with an expected CAGR of 7.1% between 2009 and 2014. The analgesics market was led by acetylsalicylic acid (representing 44.8% of the total value) followed by paracetamol and ibuprofen, with a 29.3% and 15.9% of the market share, respectively. Other analgesics accounts for the remaining 10.1% share. Bayer AG is the market leader with a 29.3% share of the market. Very interesting is the place of the widely used in Bulgaria metamizole sodium (trade name: Analgin) that gets traditionally the leading position in sales (Table 2).[11]

**Table 2.** Marketing shares of the leading analgesics and NSAIDs by trade name.[15]

	EURO sale MNF	EURO sale MNF	EURO sale MNF	EURO sale MNF
	Qtr/M03/2009	Qtr/M06/2009	Qtr/M09/2009	Qtr/M12/2009
02 PAIN RELIEF	3 570 208	3 463 125	4 456 891	4 872 480
02A GENERAL PAIN RELIEF	3 570 208	3 463 125	4 456 891	4 872 480
02A1 GENERAL PAIN RELIEF ADULTS	3 570 208	3 463 125	4 456 891	4 872 480
Analgin	974 565	1 139 589	1 004 954	1 037 045
Paracetamol	515 088	377 059	404 314	679 138
Upsarin C	401 824	206 744	300 564	643 996
Aspirin C	295 725	162 060	231 649	632 789
Acetysal	150 812	205 345	961 220	353 372
Nurofen	170 847	228 984	361 689	282 378
Benalgin	192 683	210 102	243 094	185 903
Aspetin Adipharm	31 218	8 913	166 629	172 914
Hexalgin	58 586	96 649	91 606	128 403
Aspirin	63 777	56 100	64 090	96 531

The Association of the European Self-Medication Industry (AESGP) is the official representative of the manufacturers of OTC and herbal medicines in Europe. It includes companies of different countries and Bulgaria is among them. In June 2011 the AESGP reports market sales data for the previous three years. Data for Bulgaria are pointed in the following tables (Tables 3 and 4).[12] There is a clear increase in the share of non-prescription drugs and especially in the share of analgesics and cough and cold OTC drugs for the observed period.

**Table 3.** Bulgarian non-prescription pharmaceutical market 2008–2010.

European non-prescription pharmaceutical market 2008–2010						
	At consumer price level (euro millions)			As a percentage of the total pharmaceutical market (excluding hospital sales)		
	2008	2009	2010	2008	2009	2010
Bulgaria	192	203	226	28.2	25.7	25.3

Notes: Non-prescription pharmaceutical market: sales of all medicinal products legally available without a medical prescription at public price level, including value-added tax (VAT).

**Table 4.** Self-medication product groups in Bulgaria: 2008–2010.

	Cough and cold – at consumer price level (euro millions)			Analgesics – at consumer price level (euro millions)		
	2008	2009	2010	2008	2009	2010
Bulgaria	46.0	50.0	57.0	42.0	44.0	48.0

### OTC market strategies and tools in Bulgaria

According to BMI reports of February 2010 and June 2011, the value of the pharmaceutical market in Bulgaria has increased form BGN 1.704 bn in 2008 to BGN 2.03 bn in 2011. The prognosis for the next 10 years is an increase to BGN 3.25 bn in 2019. BMI points the regional perspectives for the pharmaceutical companies by countries on the basis of Pharmaceutical and Healthcare Business Environment ratings. In the report of November 2009 Bulgaria possesses ratings of 49.4 as it is the average regional level and the country takes the 9th position. In the next BMI report of February 2010 changes can be distinguished as Bulgaria slips to the 14th position as a result of slower market recovery compared to many other countries (Table 5).[10,1,13]

**Table 5.** Regional ranking of Bulgaria for 2009–2010.[10,1]

	Limits of potential returns			Risks of realization of returns			Ranking	
	Pharmaceutical market	Country structure	Limits	Market risks	Country risk	Risks	Pharmaceutical rating	Regional ranking
11.09	40	63	46	53	57	55	49.4	9
02.10	30	63	38	53	57	55	44.9	14

### SWOT analysis

Taking in mind the characteristics of the Bulgarian market for OTC and on the basis of the results, an SWOT analysis was performed. The following facts can be pointed for the OTC and especially concerning the market of NSAIDs:

*Strengths*. Modernization and privatization of the local pharmaceutical industry; the local production benefits from rapid development in key export markets such as Russia and Ukraine; the EU membership significantly improves regulatory environment; investment in the private sector shows increase.

*Weaknesses*. The debts and mismanagement in the state hospital sector affect the entire production and drug logistic chain; pricing and reimbursement system are defined as opaque and changeable; and deep recession levels. Unfortunately, the out-of-pocket payments still present high levels. Another fact is that some wholesalers dominate with dealings with hospitals and private retail channels.

*Opportunities*. For the national healthcare system, the opportunities are as follows: increased healthcare awareness and patient demands; an ageing population and privatization of health insurance funds that should increase treatment options. It is considered that Bulgaria has one of the fastest growing drug markets in the region.

*Threats*. Payment delays; the high level of corruption and counterfeit drugs – a threat to the pharmaceutical investments. The most significant threat is that recently the Bulgarian healthcare system is ranked as the worst in Europe.

It is interesting to assess the marketing strategies of Sopharma PLC, being one of the Bulgarian manufactures at the generic and OTC market. Sopharma PLC is one of the leading representatives of pharmaceutical wholesalers and drug producers in Bulgaria. It is the second largest pharmaceutical manufacturer in Bulgaria and it is one of the country's oldest companies as being established in 1933.[10,14] It has been privatized in 2000 and merged with another national drug producer Unipharm. Their current market share is approximately 20% which presents the largest share of incomes. The product portfolio includes products of different therapeutic groups including NSAIDs and analgesics. An interesting moment in the history and activity of the company is the establishment of Sopharma Trading JSC in 2006. It is defined as a successful project of Sopharma PLC through which the vertical integration is accomplished. All of its activities are according to the guidelines for Good Distribution Practice and at the same time it offers marketing management services to its clients. The company provides market analyses and data, product positioning and advertisement, merchandising and promotional actions. According to surveys few important threats are pointed: need for sources of capital for further growth; and the counterfeit drug market in Bulgaria and the region. Sopharma takes third place in the classification of leading pharmaceutical companies by sales in the country in 2009.[10,1]

One of the products of Sopharma PLC – Analgin (metamizole sodium) – traditionally is the most sold analgesic in Bulgaria for more than 10 years. Its leading position is clearly demonstrated in Table 2. The positioning of Analgin is impossible in many countries, where the use of the active substance metamizole sodium is prohibited due to the possibility of agranulocytosis.

The leading position of Analgin is distinguished in the following groups and periods:

1. Top ten products in sales in value for the last quarter in 2009;
2. Top products in sales in units for the months January–May 2010;
3. Top products in sales in value for the months January–May 2010.

Sopharma PLC successfully expands its activity abroad through subsidiary companies. Its production is influenced by the political and economic situation as a whole in Central and Eastern Europe. The producer also wins positions in foreign markets. The results from the SWOT analysis of Sopharma PLC are shown as follows:

Strengths:

1. Well-established manufacturing basis in Bulgaria;
2. Subsidiary companies;
3. GMP and ISO certification;
4. Strong position on the Russian market.

Weaknesses:

1. Serbian market – as the most promising among the Central European and Eastern European countries;
2. Decrease in the market grow at the Russian market.

Opportunities:

1. Limited local market threat;
2. Vertical integration with wholesalers;
3. Possibilities for expansion through acquisition.

Threats:

1. Way of finding of attractable sources of funds for future acquisitions;
2. The counterfeit drug market in the country and the region.

## Conclusions

The study results show that the legislation frame in Bulgaria gives us an opportunity for regulation of the NSAID market in order to avoid incorrect marketing approaches such as disloyal competition. The collected data confirm the fact that in recent years there is an increase of the OTC consumption. The Bulgarian producers of these drugs are well acquainted with the marketing strategies and are properly applying them. The threats that they are facing include additional financial resources and measures in order to restrict the counterfeit NSAID drug market, the leading threat for pharmaceutical markets worldwide. In July 2011, the EU strengthened the protection of patients and consumers by adopting a new Directive on counterfeit medicines for human use. This Directive aims to prevent counterfeit medicines to enter the legal supply chain and reach patients. It applies new measures such as obligatory features on the outer packaging of medicines to demonstrate that they are authentic; requirements for the inspection of the manufacturers of pharmaceutical ingredients; obligation for manufacturers and distributors to report any suspicion of counterfeit medicines; and obligatory logo that must be placed on the websites of legally operating online pharmacies, with a link to official national registers. A successive measure for the Bulgarian manufactures and wholesalers will be the implementation of the new Directive in the Bulgarian drug law.
